# Supplementary material for: Loss of the molecular clock in myeloid cells exacerbates T cell-mediated CNS autoimmune disease
Source: Nat Commun. 2017 Dec 12;8:1923. doi: 10.1038/s41467-017-02111-0 (PMC5727202; doi:10.1038/s41467-017-02111-0)
Supplement: Supplementary file 1 — Supplementary Information [file 41467_2017_2111_MOESM1_ESM.pdf]

Supplementary Figure 1

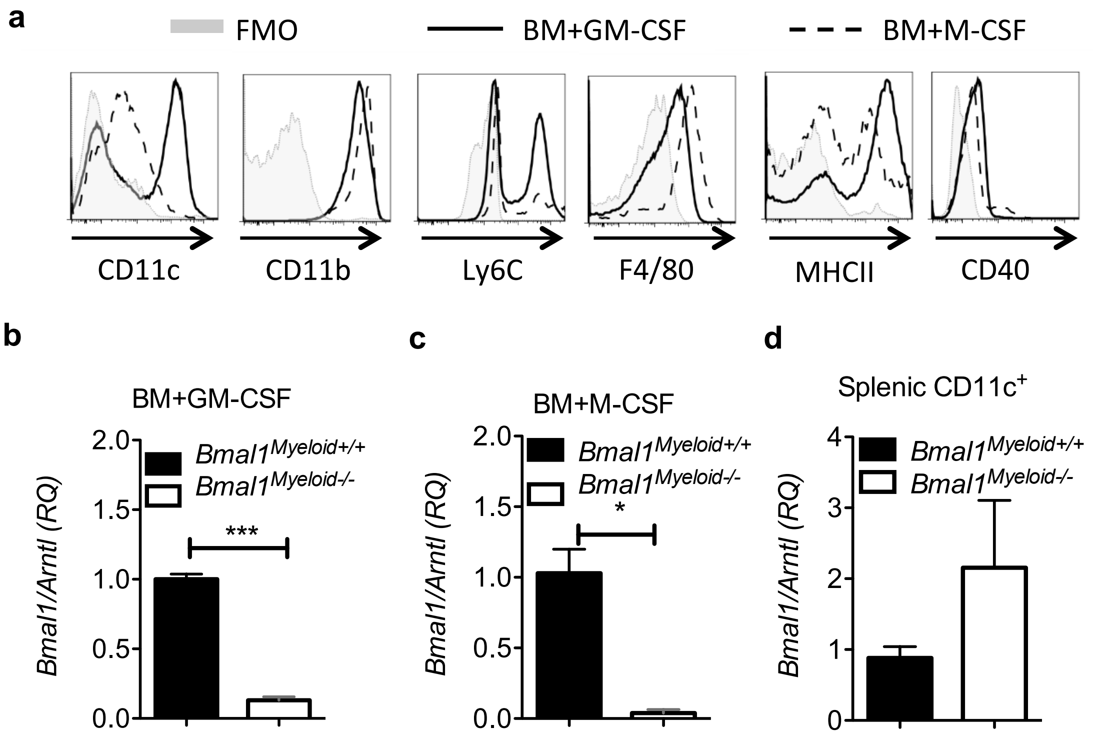

Bone marrow expanded cells from *Bmal1*$^{Myeloid-/-}$ mice are devoid of *Bmal1*.

Bone marrow (BM) cells from C57BL/6 mice (a) or *Bmal1*$^{Myeloid+/+}$ and *Bmal1*$^{Myeloid-/-}$ mice (b-d) was cultured in the presence of granulocyte-macrophage colony-stimulating factor (GM-CSF) (20 ng/ml) or macrophage colony-stimulating factor (M-CSF) (100 ng/ml) for 6 d. (a) FACS surface staining for CD11c, CD11b, Ly6C, F4/80, MHCII or CD40. (b and c) Cells were harvested into Trizol and RNA was extracted. RT-PCR for *Bmal1* was performed on each sample, relative to *Bmal1*$^{Myeloid+/+}$ cells.

(d) CD11c^{+} cells were FACS-sorted from the spleens of naïve *Bmal1*$^{Myeloid+/+}$ and *Bmal1*$^{Myeloid-/-}$ mice. RNA was extracted by Trizol from sorted cells and was tested by RT-PCR for Bmal1 (n=3). FMO = Flourescence minus one. RQ = Relative Quantification. Presented as means +/- standard error of the mean (SEM). Statistics were performed by Mann-Whitney U test. *, p<0.05; ***p<0.001.

Supplementary Figure 2

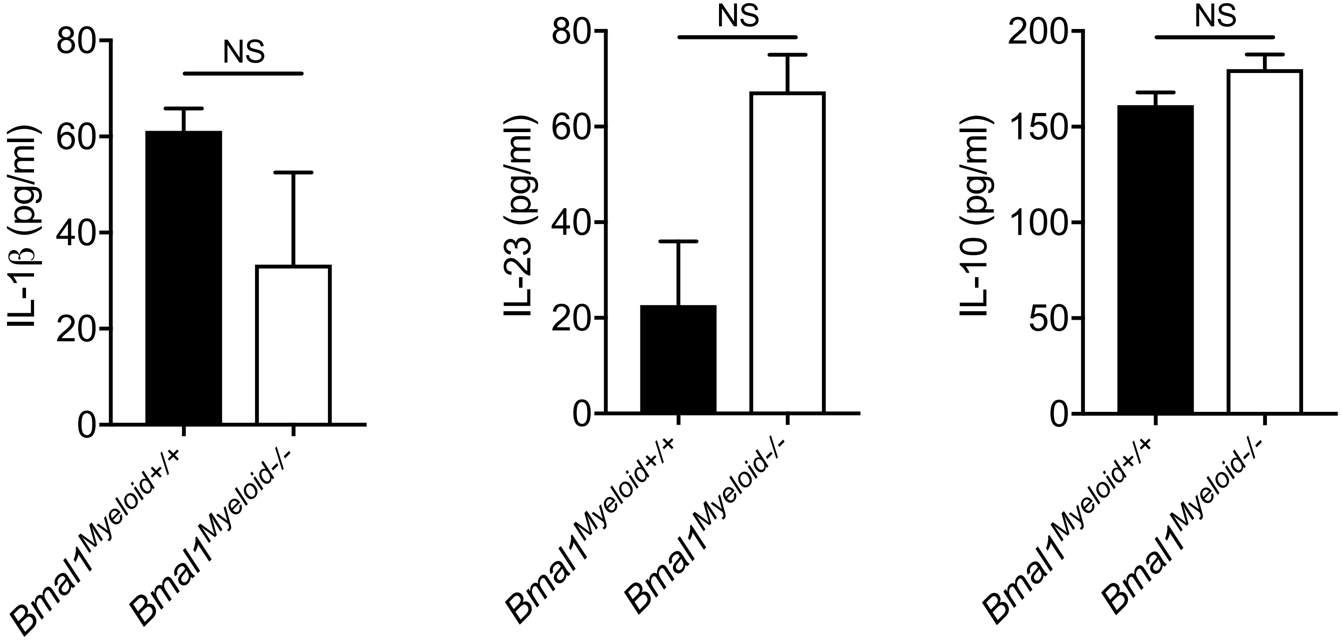

Loss of *Bmal1* from bone marrow cells cultured with M-CSF does not affect MTB driven pro-inflammatory responses.

Bone marrow (BM) cells isolated from *Bmal1*$^{Myeloid+/+}$ or *Bmal1*$^{Myeloid-/-}$ mice were cultured with macrophage colony-stimulating factor (M-CSF) (100 ng/ml). After 6 days cells were harvested and stimulated with *Mycobacterium tuberculosis* (MTB) (100 µg/ml). Supernatants were removed at 24 h and tested by ELISA for cytokine production (n=3). Presented as means +/- standard error of the mean (SEM). Statistics were performed by Mann-Whitney U test.

Supplementary Figure 3

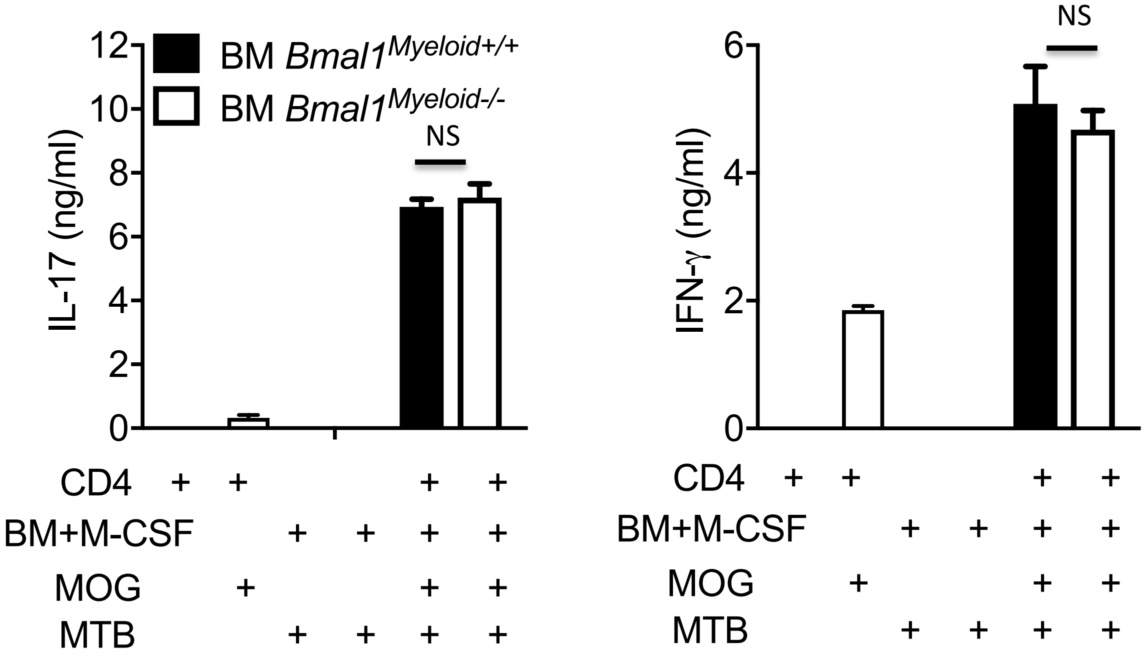

Loss of *Bmal1* from bone marrow cells cultured with M-CSF does not affect antigen-specific pro-inflammatory T cell responses.

Bone marrow (BM) cells from $Bmal1^{Myeloid+/+}$ and $Bmal1^{Myeloid-/-}$ mice were cultured in the presence of macrophage colony-stimulating factor (M-CSF). After 6 d cells were harvested and incubated with myelin oligodendrocyte glycoprotein (MOG_{35-55}) (25 µg/ml) + with *Mycobacterium tuberculosis* (MTB) (100 µg/ml) or with medium for 3 h prior to the addition of MACS purified CD4 T cells isolated from the draining lymph nodes of 7 d MOG_{35-55}+ complete Freund's adjuvant (CFA) immunised $Bmal1^{Myeloid+/+}$.

After 72 h of co-culture supernatants were removed and tested for IL-17 and IFN-γ by ELISA (n=3). Presented as means +/- standard error of the mean (SEM). Statistics were performed by Mann-Whitney U test.

Supplementary Figure 4

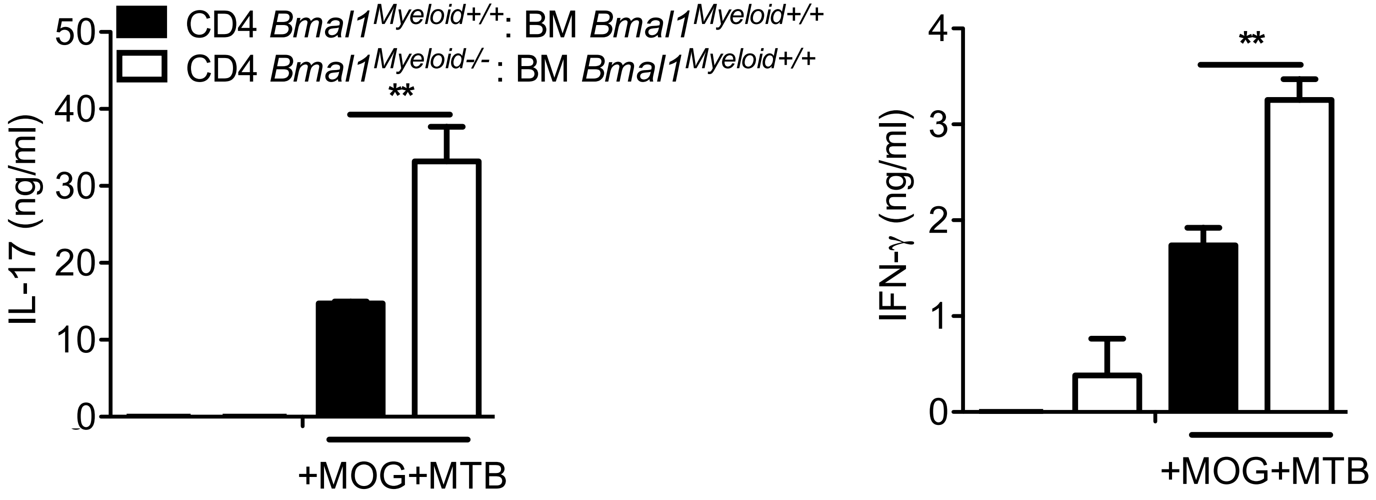

T cells isolated from immunized mice lacking myeloid *Bmal1* display enhanced antigen specific pro-inflammatory responses *ex vivo*.

Bone marrow (BM) cells from *Bmal1*$^{Myeloid+/+}$ was cultured in the presence of granulocyte-macrophage colony-stimulating factor (GM-CSF). After 6 d cells were harvested and incubated with myelin oligodendrocyte glycoprotein ($MOG_{35\text{-}55}$) (25 µg/ml) + *Mycobacterium tuberculosis* (MTB) (100 µg/ml) or with medium for 3 h prior to the addition of MACS purified CD4 T cells isolated from the draining lymph nodes of 7 d $MOG_{35\text{-}55}$+ complete Freund's adjuvant (CFA) immunised *Bmal1*$^{Myeloid+/+}$ or *Bmal1*$^{Myeloid-/-}$ mice. After 72 h of co-culture supernatants were removed and tested for IL-17 and IFN-γ by ELISA (n=3). Presented as means +/- standard error of the mean (SEM). Statistics performed by Mann-Whitney U test. **, p<0.01.

Supplementary Figure 5

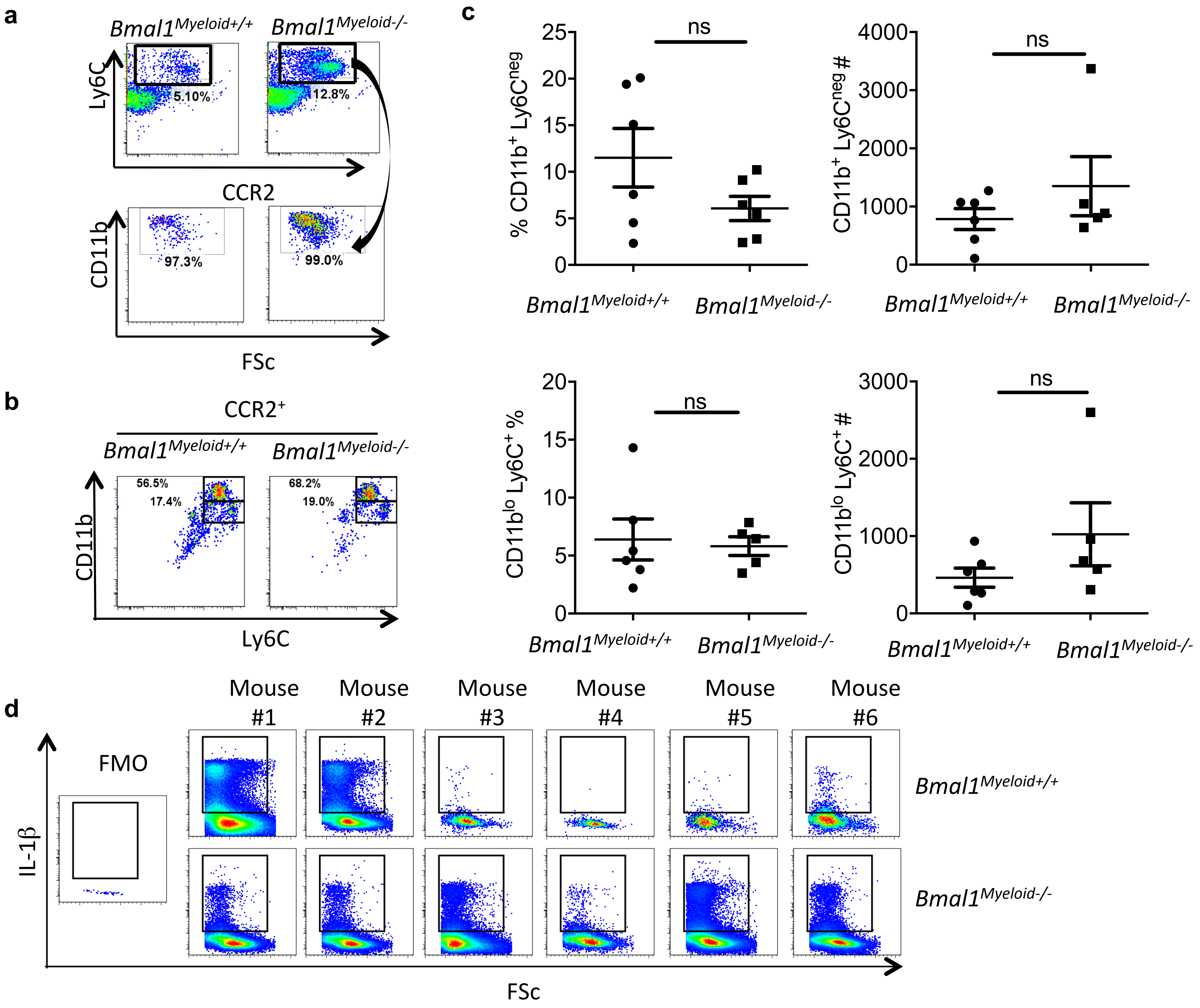

Inflammatory populations associated with disease progression are enhanced in *Bmal1*$^{Myeloid-/-}$ mice at the height of EAE.

Mice were immunised with myelin oligodendrocyte glycoprotein (MOG_{35-55}) + complete Freund's adjuvant (CFA), and injected with pertussis toxin (PT) (125 ng/mouse) on d 0 and d 2. (a-c) 14 d post immunisation infiltrating mononuclear cells in the spinal cords of mice and were stained on their surface for a CD11b, Ly6C and CCR2 expression. (a) Percentage of CD11b$^+$ cells gating on Ly6C$^+$CCR2$^+$ cells the percentage of CD11b$^+$ cells v FSc. (b) Percentage of CD11b^{hi}Ly6C$^+$ of CCR2$^+$ cells were examined. (c) Numbers and percentages of CD11b$^+$ cells were examined (n=5-6). (d) D 10 post immunisation for EAE, IL-1β expression was determined in mononuclear cells isolated from the brains of 6 individual *Bmal1*$^{Myeloid+/+}$ or *Bmal1*$^{Myeloid-/-}$ mice, gating on live CD45$^+$CD11b$^+$Ly6C$^+$ cells. Presented as means +/- standard error of the mean (SEM). Statistics performed by Mann-Whitney U test.

Supplementary Figure 6

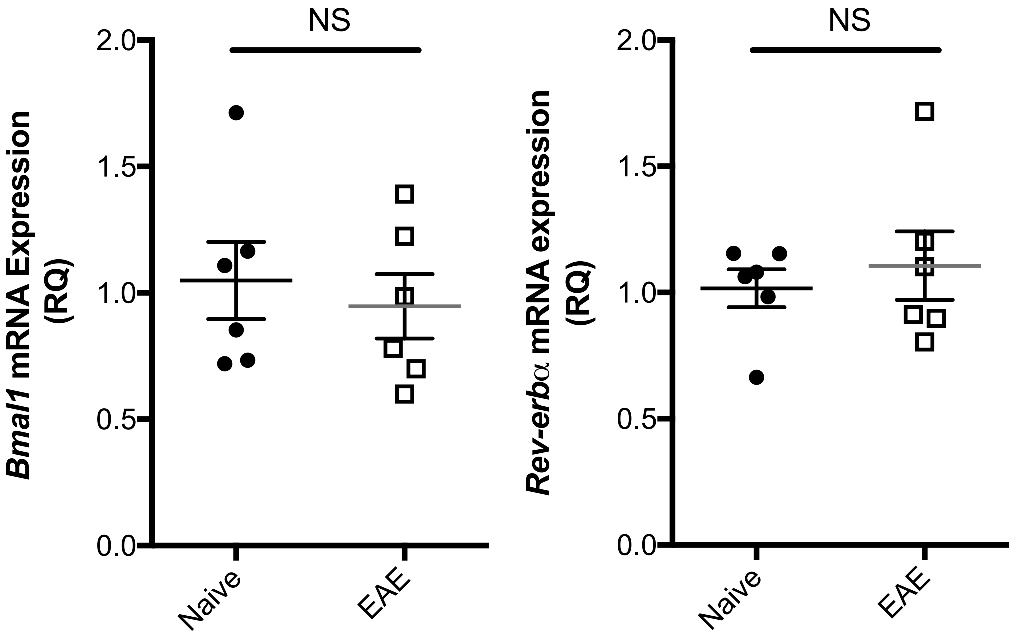

***Bmal1* and *Reverba* are not affected under systemic inflammation in spinal cords.**
Spinal cords were removed from 14 d complete Freund's adjuvant (CFA) and pertussis toxin (PT)-immunised or naïve female C57BL/6 mice (no myelin oligodendrocyte glycoprotein (MOG) was used). RT-PCR analysis of *Bmal1* and *Rev-Erba* mRNA of isolated spinal cords (n=6). RQ = Relative Quantification. Presented as means +/- standard error of the mean (SEM). Statistics performed by Mann-Whitney U test.

Supplementary Figure 7

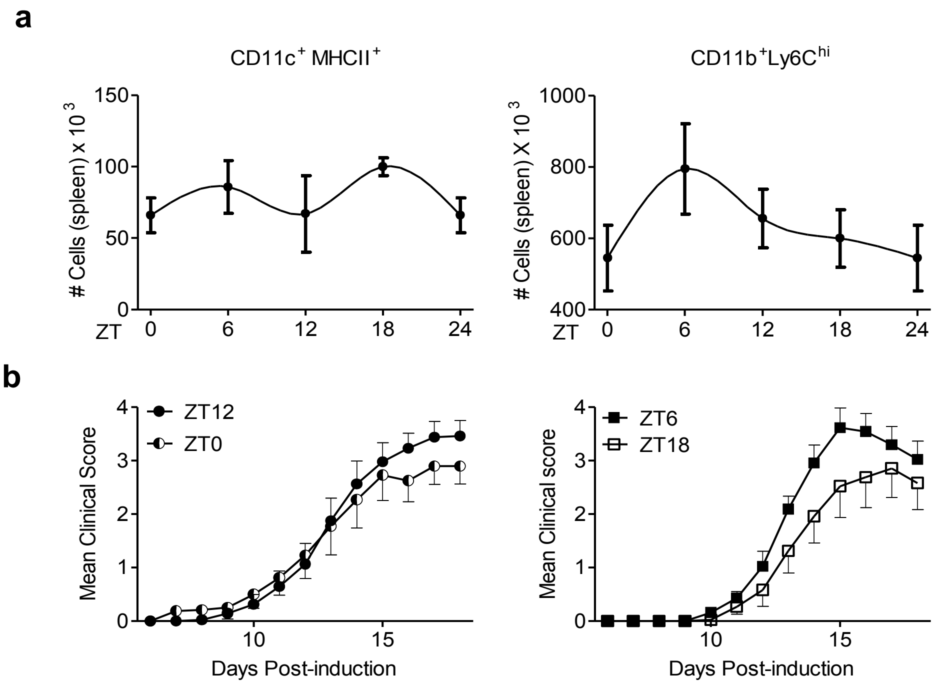

Time of day dependent alterations in the spleen and induction of Experimental Autoimmune Encephalomyelitis (EAE)

(a) CD11c$^+$MHCII$^+$ and CD11b$^+$Ly6C^{hi} populations were FACS sorted from spleens of 5 individual C57BL/6 mice at ZT0, 6, 12 or 18. ZT0 has been double plotted to continue trend. Total numbers of cells sorted were recorded for each population (n=5-6). (b) Time of day was adjusted in C57BL/6 female mice (Harlan, UK), using a light cabinet so that both ZT6 and ZT18 corresponded to 3pm and ZT0 and ZT12 corresponded to 9am. Mice were immunised with myelin oligodendrocyte glycoprotein ($MOG_{35\text{-}55}$)+ complete Freund's adjuvant (CFA) and pertussis toxin (PT) (200 ng/mouse), which had been emulsified 1 week in advance at 4pm, and mice were given a second dose of PT at 4pm or 9am on d 2. Mice were scored daily for the disease score. Data presented as means +/- standard error of the mean (SEM) of 12 mice per group.

Supplementary Figure 8

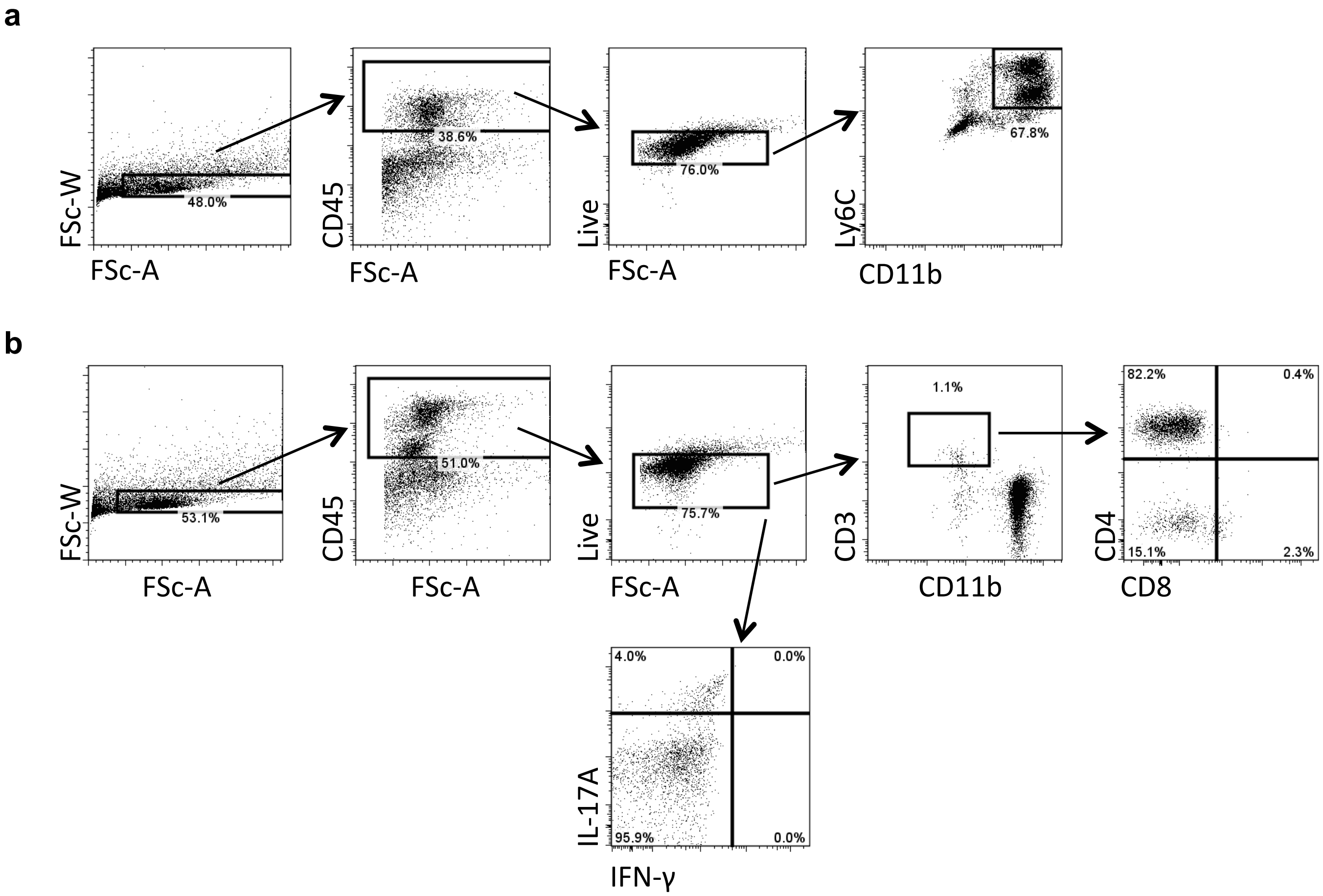

Gating strategies used for flow cytometry analysis of CD11b^{+}, CD4^{+}, CD8^{+} cells and IFN-γ^{+} and IL-17^{+}-producing cells. (a) Gating strategy used to gate on CD11b^{+} cells as presented on Figure 3. The same type of gating strategy was used in Figures 1, 2 and 6 (b) Gating strategy used to gate on CD4^{+} , CD8^{+} cells and IFN-γ^{+} and IL-17^{+}-producing cells as presented in Figure 4.
